# Topical and Intralesional Treatments for Skin Metastases and Locoregionally Advanced Melanoma

**DOI:** 10.3390/cancers17010067

**Published:** 2024-12-29

**Authors:** María Criado-Otero, María Navedo-de las Heras, Elia Samaniego-González

**Affiliations:** Dermatology Department, Complejo Asistencial Universitario de León, 24008 León, Spain; mnavedo@saludcastillayleon.es (M.N.-d.l.H.); esamaniego@saludcastillayleon.es (E.S.-G.)

**Keywords:** locoregionally advanced melanoma, skin metastases, satellite metastases, in-transit metastases, topical treatment, intralesional treatment, Diphenylcyclopropenone, imiquimod, 5-FU, Bacillus Calmette–Guerin, IL-2, PV-10, Talimogene Laherparepvec, electrochemotherapy

## Abstract

Cutaneous melanoma is a malignant neoplasm with local and distant metastatic potential. When feasible, surgery is the first line of treatment in locoregionally advanced disease. Topical and intralesional treatments can be an alternative second-line treatment. The aim of this article was to perform a narrative review of the most widely used topical and intralesional treatments for locoregionally advanced melanoma. Diphenciprone, imiquimod and 5-florouracil were included as topical treatments and bacillus Calmette-Guerin, interleukin 2, rose bengal, talimogene laherparepvec and electrochemotherapy were included as intralesional treatments. Brief comments on other alternatives in development such as interferon-alpha, interleukin-12, ipilimumab and intralesional daromun are presented. Topical treatments generally have higher response rates in epidermal metastases than in deeper metastases. In addition, the larger the lesions, the worse they tend to respond to local treatments. Some reports show that combining certain systemic treatments and topical or intralesional therapies can improve response rates. It has also been described in a few papers that non-injected lesions may respond after the application of a local therapy in distant skin-metastases. Many of these intralesional treatments are being combined in different investigations with systemic immunotherapies, with the aim of obtaining synergic responses in those patients with refractory disease.

## Simple Summary

Melanoma is a common and aggressive malignant skin tumor with the capacity to metastasize in both internal organs and skin. It has traditionally been resistant to conventional systemic chemotherapeutic drugs. With the development of systemic immunotherapy and new targeted therapies, the management and prognostic paradigm for advanced melanoma has changed. Along with the development of these new therapeutic agents, topical and intralesional treatments for metastatic cutaneous melanoma have also emerged and may be an alternative to surgical management when surgery is not feasible. The selection of the most adequate approach depends on the availability of the different treatments, the size of the lesion, the stage of the disease, the characteristics of the patient and the physician’s experience and knowledge. In this narrative review, we focus on the outcomes offered by different topical and intralesional therapies for cutaneous metastatic melanoma, alone or in combination with systemic therapies.

## 1. Introduction

Melanoma is a malignancy that, due to its resistance to cytotoxic chemotherapy, remained for years outside of advances in oncologic treatments. For this reason, it was one of the first targets for the study of local and intralesional therapies [1]. The basis for the development of these treatments is the proven recognition of tumor cells by the immune system and the fact that the development of a strong immune response against them is associated with more favorable clinical outcomes. This natural immune response, which is by definition not sufficient in patients with progressive or metastatic disease, can be stimulated locally with different intralesional and topical therapies, combined or independent of each other and combined or independent of systemic treatments [2].

The incidence of melanoma has increased in the last few years with an estimated worldwide number of new cases between 325,000 and 57,000 deaths due to melanoma in 2020. Predictions suggest that the incidence will continue to rise over the next two decades [3]. The prognosis of patients with melanoma is highly variable depending on their stage. Metastatic melanoma, despite new targeted therapies with BRAF and MEK inhibitors as well as immunotherapy with CTLA-4, PD-1 and PD-L1 inhibitors, continues to have high mortality rates, but there have been significant advances compared to previous years [4,5].

Within metastatic melanoma, we must differentiate melanoma with skin metastases from melanoma with visceral metastases, as their prognosis and treatment are considerably different. Skin metastases in melanoma are relatively frequent and can be a sign of recurrence or of advanced disease. Depending on the distance of skin metastases from the primary tumor, classically, a distinction has been made between satellite metastases (SMs), defined as those located up to 2 cm from the primary tumor, and in-transit metastases (ITMs), which are those that develop between 2 cm from the primary tumor and the first lymph node drainage territory. Both of them entail lymphatic extension, resulting in locoregionally advanced disease. Distant metastases, which are those that appear in any area of the cutaneous integument far from the primary tumor as a result of hematogenous dissemination, are the third type of skin metastases that can be found [6]. It is important to take these differences into consideration, since in the eighth edition of the *American Joint Committee on Cancer* (AJCC) melanoma staging system [7], the presence of distant skin metastases (M1a) is considered stage IV, while satellite metastases and ITMs are considered stage III, corresponding to N1c when they appear in isolation without the associated lymphatic invasion. These distinctions have implications in terms of prognosis. In the eighth edition of AJCC analyses, patients with stage III had widely variable prognosis, ranging from 93% 5-year melanoma-specific survival for stage IIIA to 32% for stage IIID disease. Patients with stage IV melanoma have historically had poor prognosis with median survival from time of initial stage IV diagnosis of 6–7.5 months and a 5-year survival of <10% [8].

Surgical treatment with adequate margins is, until today, the treatment of choice for skin metastases of melanoma, which is performed with curative intentions [9]. In some cases, due to the anatomical location, the patient’s comorbidity or the existence of advanced disease, surgical treatment is not feasible. In these circumstances, the topical and intralesional therapies that will be discussed in this article can be appropriate therapeutic alternatives.

An extensive review has recently been published in this journal that includes the most relevant clinical trials from phase II onwards for various types of intralesional treatments: xhantene dyes, cytokines, antibody–cytokine fusion proteins, oncolytic viral therapies, toll-like receptor agonists and others as well as future perspectives [10]. For this reason, the objective of this article was to conduct a narrative review of the most relevant currently available topical and intralesional therapies for the treatment of locoregionally advanced melanoma with SM or ITM (N1c, N2c or N3c according to the 8th edition of AJCC), as well as distant cutaneous metastases.

## 2. Materials and Methods

We performed a bibliographic search using PubMed, Dialnet and Google Advanced Search with terms related to the objective such as “Skin Metastases of Melanoma”, ‘Advanced Melanoma’ combined with ‘Topical Treatment’, ‘Intralesional Treatment’, ‘Diphenylcyclopropenone’, ‘Imiquimod’, ‘5-FU’, ‘Bacillus Calmette-Guerin’, ‘IL-2’, ‘PV-10’, ‘Rose Bengal’, ‘Talimogene Laherparepvec’ and ‘Electrochemotherapy’. Randomized clinical trials were prioritized in the selection of articles and those that provided data in terms of overall survival and relapse-free survival, with complete and partial responses. The selection of articles was performed independently by two dermatologists through careful reading of the titles and abstracts. This was followed by a review of the references of the most relevant papers to identify other publications that could be included in this article. The selection of articles and the analysis of the most relevant data were reviewed by a third dermatologist that specialized in cutaneous oncology.

## 3. Topical Treatments

### 3.1. Diphenylcyclopropenone

Diphenylcyclopropenone (DPC) is a potent contact sensitizer. Damian et al. [11] first described its use in metastatic cutaneous melanoma in 2007. To be used for the treatment of melanoma, the patient is first sensitized to the compound via the application of 2% DPC in acetone in a Finn chamber, occluded for 48 h. With this procedure, epicutaneous sensitization is achieved after 8–15 days. This is followed by the application of DPC to the skin lesion, diluted in an appropriate aqueous cream at a concentration between 0.01 and 0.1%, without occlusion, once every 24 h, for at least one month unless intolerance occurs. Damian et al. subsequently published a series of 50 patients with cutaneous metastatic melanoma treated with DPC with good results, especially in thin lesions involving only the epidermis or superficial dermis [12]. The complete response rate was 46%, maintained for a mean of 17 months.

Later, Veverka et al. [13] published a series of 13 patients treated with topical DCP in combination with other treatments (imiquimod, dabrafenib, ipilimumbab, pulsed-dye laser and GM-CSF). They were unable to reproduce the results previously published by other scientists, finding that their cohort of patients treated exclusively with DPC had progression of cutaneous disease. Other published case series showed variable and hardly comparable results, as the characteristics of the patients treated and the follow-up time differed. In any case, the complete response rates were worse than in Damian et al.’s work, with complete response in 13 to 22% of the patients [8,13,14,15,16]. Most studies suggest that DPC shows better results in thin or epidermotropic melanoma lesions, either for primary melanomas or cutaneous metastases of melanoma [8,14,15]. Some studies have also shown better results in lesions on the lower extremities in women [8], although the quality of the evidence is questionable because there are case series with a limited number of patients. A very interesting paper was published by Ci Lo et al. [17]. In this work, they conducted a retrospective study comparing isolated limb infusion and the application of DPC. The most notable finding of this study is that the patients, who experience progression at some point during DPC treatment with lesions located on a limb that are then treated with ILI, show an increase in progression-free survival. Although we have not found any studies specifically addressing the combination of DPC treatment with immunotherapy or other systemic treatments, the paper published by Veverka et al. [13] reported several patients in these circumstances, with no significant adverse effects reported with the combination of both strategies. The most representative studies in this topic, as well as their results, are shown in Table 1.

The studies reviewed in this article show that DPC is a useful treatment in patients with cutaneous metastases of melanoma who are not candidates for other treatments and also suggest that its combination with other immunotherapies or systemic treatments may provide synergistic effects. These conclusions are supported by the meta-analysis performed by Lôbo et al. [18] on the response rates of skin melanoma metastases treated with DCP, in which they found a complete response rate with a random effects model of 29.94% (95% confidence interval, 20.65–41.22) and an overall response rate (equal to complete response plus partial response) with a random effects model of 60.48% (95% confidence interval, 45.90–73.42).

### 3.2. Imiquimod

Imiquimod is a toll-like receptor (TLR) 7 and 8 agonist molecule that stimulates dendritic cell activity and increases the Th1 and cytotoxic CD8 immune response. This immune activation leads to an antitumor response [6]. There is evidence of its effectiveness in some cases of non-surgical lentigo maligna [19,20]. For advanced melanoma, a few clinical trials, case series and single case reports of patients with cutaneous metastases of melanoma have been published showing a response to topical imiquimod alone or in combination with other treatments, but the available evidence is generally scarce, leading to its consideration as a second-line treatment. The results are better in superficial lesions affecting as far as the dermis and worse if they involve the subcutaneous cellular tissue [21,22], although there are some cases that described a satisfactory response in subcutaneous lesions [23]. Inflammation and local adverse effects may lead to the discontinuation of treatment due to intolerance, especially in elderly or fragile patients.

There are some trials combining topical imiquimod with intralesional IL-2 injection. Green et al. [22] published a phase I/II clinical trial combining intralesional IL-2 injection with topical imiquimod application for 4 weeks in 13 patients with ITM that were resistant to other treatments. They obtained a complete response rate in 74 of the 182 lesions (40.7%) with good tolerance. In addition, Shi et al. [24] performed a retrospective study associating intralesional IL-2, topical imiquimod and retinoids in 11 patients. The response was complete in all of them, with no recurrence after 24 months. These results are similar to the ones reported in three patients by Shirakawa-García et al. [23]. Although these data are excellent, a larger trial would be necessary to confirm the role of retinoids as adjuvant therapy in a therapeutic protocol of topical imiquimod plus intralesional IL-2.

In addition to this combined approach, there is another study published by Rivas-Tolosa et al., combining topical imiquimod with cryotherapy, which reported complete response in 40% of patients and partial response in 25%. Alternatively, topical imiquimod combined with monobenzone (a depigmenting agent) has been used in a phase II clinical trial with a total of 21 patients by Teulings et al. [25], showing regression of cutaneous metastases in 38% of the patients at an initial stage, which increased to 52% after a longer course of therapy.

Imiquimod has also been combined in a few cases with gentian violet [26], topical 5-FU [27], systemic ipilimumab [28] cryotherapy [29,30], carbon dioxide laser and electrocautery [31], intralesional Bacillus Calmette-Guérin (BCG) [32,33], intralesional talimogene laherparepvec (T-VEC) [34] and brachytherapy [35], demonstrating a promising synergy with these treatments, although larger-scale studies would be necessary to determine their role.

Although the number of isolated cases of locoregionally advanced melanoma with cutaneous metastases that responded to imiquimod alone or in combination is remarkable, it is not considered the sole routine therapeutic alternative for this patient profile. The most remarkable studies versing about imiquimod and its combinations for the treatment of locoregionally advanced melanoma with cutaneous metastases are synthesized in Table 2.

### 3.3. 5-Florouracil

The 5-florouracil (5-FU) is an antimetabolite commonly used as a topical treatment for non-melanoma skin cancers and precancerous skin lesions [36]. It inhibits DNA synthesis by inducing apoptosis of the malignant cells. It is not typically prescribed as a treatment for locoregionally advanced melanoma, although Florin et al. [27] reported promising results with a combined regimen of topical 5-FU and topical imiquimod. They reported clinical response in 98% of the lesions treated, with complete response in 56% of them. The rest of the lesions (2%) remained stable. None of the subcutaneous lesions achieved complete response, hypothetically due to a lack of deep skin penetration of the topical treatments. The tolerance to this combined treatment was good, although all complete responses were observed after ulceration of the skin. This study is also included in Table 2.

Regarding the level of scientific evidence reported on the use of 5-FU in melanoma with cutaneous metastases, we consider that it should be reserved for thin lesions which do not affect subcutaneous cellular tissue. It can be used when the decision to use topical imiquimod has been made, and there is a desire to complement the treatment with another topical agent.

## 4. Intralesional Treatments

### 4.1. Bacillus Calmette–Guerin (BCG)

The intralesional injection of BCG for the treatment of locoregionally advanced melanoma was first performed by Dr. Donald L. Morton [1]. After the successful treatment of a patient with in-transit metastases that involved her arm and who refused amputation, he published a series of 151 patients treated with intralesional immunotherapy with BCG in the 1970s [37]. Although the understanding of the mechanics of immunotherapy at that time was rather rudimentary, this first cohort of patients created the basis for the development of the intralesional immunotherapies that are still used until this day. Although a clinical response was obtained in many cases, Morton and other investigators who used intralesional BCG reported adverse effects such as febrile syndromes, hepatitis, pneumonitis and disseminated intravascular coagulation even with a fatal outcome [38,39,40]. This is one of the reasons why, despite being a revolutionary treatment at the time, it has now been replaced in the majority of cases by new intralesional immunotherapies, which offer better outcomes with less adverse effects.

More recently, Da Gamma et al. [41] attempted to conduct a clinical trial based on the hypothesis that the combination of systemic administration of ipilimumab (anti-CTLA-4) with intralesional BCG in patients with metastatic melanoma could optimize the antitumor immune response. The trial had to be stopped after only five patients had been treated as severe immune-mediated adverse effects were observed, with no associated clinical response. The results of these studies are summarized in Table 3.

### 4.2. Interleukin-2 (IL-2)

IL-2 is a cytokine that acts by activating several pathways that modulate lymphocyte proliferation, activity and survival through binding to the IL-2 receptor. Normally, IL-2 is produced in the physiological activation of T lymphocytes and NK cells [2]. Its ability to stimulate the immune response has allowed for its use as an intralesional treatment in melanoma with cutaneous metastases; however, its systemic administration has been discarded due to the large number of adverse effects that appear at therapeutic doses. In general, its intralesional use is well tolerated [43]. The first investigators who reported regression of skin metastases of melanoma after peritumoral injection of IL-2 were Gutwald et al. [44] in 1994. Later, Radny et al. [45], Dehesa et al. [46], Weide et al. [47] and Khoury et al. [48] published several studies supporting its antitumor effects and its therapeutic usefulness in patients with skin metastases of melanoma. Radny and his collaborators, as well as Weide and his colleagues, used intralesional IL-2 two to three times a week for several weeks continuously, obtaining a complete response between 62.5% and 78.7%. However, Khoury et al. injected IL-2 every two weeks up to at least four times and obtained a complete response in 44.6% of patients. It is interesting that the latter group of investigators observed an abscopal effect in 20.7% of patients with complete response, something that Weide et al. did not observe. Dehesa obtained very good results with IL-2 injection and was able to surgically remove several lesions that were previously too large for this approach. The results of these three trials are synthesized in Table 4.

The combination of IL-2 with other systemic or intralesional treatments is currently the subject of several studies. The combined use of intralesional IL-2 with checkpoint inhibitors has been described in patients with metastatic melanoma resistant to the use of checkpoint inhibitors alone. Rafei-Shamsabadi et al. [49] published a series of nine patients with this profile of resistance to PD-1 inhibitor monotherapy. Three patients showed a complete response; three patients showed a partial response, and three patients showed disease progression upon receiving this combination therapy. Although the patient sample was small, it suggested a favorable synergy between both treatments.

In 2017, a phase II clinical trial (NTC01480323) tested the combined use of systemic ipilimumab and intralesional IL-2 and found that the results of the combined therapy were not superior to those of ipilimumab alone [50].

There is also a recently published study combining intralesional IL-2 with intralesional BCG in patients with stage III and IV melanoma and skin metastases with positive results and good tolerance, showing a complete response in 60%, progressive disease in 20% and no response in 20% of the patients. The overall response rate was 70% with a median overall survival of 35.5 months [51].

Further investigations are also being carried out with the aim of improving the response achieved by using recombinant fusion proteins and linking cytokines with known activity, such as IL-2 and tumor necrosis factor to an engineered antibody fragment to localized tumor cells. For example, there is a phase II study (NCT06284590) currently under development that aims to evaluate the efficacy of the single agent L19IL2, single agent L19TNF, and the combination of L19IL2 + L19TNF given concurrently with anti-PD1 therapy.

In summary, treatment with intralesional IL-2 is well tolerated and has good response rates. Some studies have shown better results in stage III patients and in metastases located in the dermis than in stage IV melanoma patients and subcutaneous lesions [47]. One of the main drawbacks for this treatment is the frequency of its administration (2–3 times per week), which could be reduced with the synergic combination with other treatments, for which there is still a need for further research to establish specific guidelines [6]. The dose administered and the number of sessions differ among the different trials and make it difficult to standardize the results.

### 4.3. Rose Bengal (PV-10)

PV-10 is a preparation of Rose Bengal (xhantene dye) at 10% concentration in saline. It is a chemical compound that demonstrated its antitumor capacity initially in mice and in investigations concerning its possible use in photodynamic therapy [2,10]. The hypothesis about its mechanism of action is that it causes the release of photolytic lysosomal enzymes that cause tumoral lysis. The lysis of tumor cells leads to the hypothetical exposure of their antigens and the activation of the immune response [6]. Furthermore, it has been found in vitro that PV-10 induces the selective lysis of melanoma cells but not of normal fibroblasts [10].

Several studies have been conducted using intralesional PV-10 as a treatment for cutaneous metastases of melanoma. Thompson et al. [52] published the first data from a phase II clinical trial in which they reported a complete response rate of 26% and an overall response rate of 51% with a median duration of response of 4 months. Some uninjected lesions also regressed. This first trial suggested better results in patients who experienced blistering of the lesions after injection, but this could not be corroborated by Read et al. [53] in a later published paper. In their study they found no statistically significant factors that correlated with a better response to intralesional PV-10 therapy. A paper by Lippey et al. [54] also reported similar results in terms of response rates to those obtained by Thomson and his colleagues *(*Table 5*).*

In addition, results from a series of patients treated with a combination of intralesional PV-10 with radiotherapy have been published with an overall response rate of 86.6% and good tolerance [55], although this is a single-arm study, and the quality and quantity of the scientific evidence are limited. Also, a clinical trial comparing the administration of intravenous pembrolizumab alone versus intravenous pembrolizumab and intralesional PV-10 is also in progress, with the primary outcome of assessing the development of adverse effects and the impact on progression-free survival rates [56].

Intralesional PV-10 treatment has the advantage of being relatively well tolerated and requiring a low number of injections before a therapeutic response is achieved. The possibility of combining it with systemic therapies such as pembrolizumab is a promising prospect for the future.

### 4.4. Talimogene Laherparepvec (T-VEC)

Talimogene laherparepvec (T-VEC) is a genetically modified virus derived from an attenuated strain of herpes simplex virus type 1. It has been engineered to selectively induce the lysis of cancer cells, stimulate local inflammation and enhance antigen presentation, thereby driving immune responses against tumor cells [57]. The virus contains deletions of two genes, ICP34.5 and ICP47, and incorporates the genes for granulocyte–macrophage colony-stimulating factor (GM-CSF) and US11. The absence of ICP34.5 allows for viral replication specifically within cancer cells, while the deletion of ICP47 prevents the downregulation of cancer cell antigens following viral infection. This enables the virus to replicate within tumor cells, causing their destruction and the release of various tumor-associated antigens [58]. The inclusion of GM-CSF enhances the immune response against the tumor via the recognition of the newly released tumor antigens. Intralesional T-VEC has received FDA and EMA approval in 2015 based on the results of the OPTiM phase III trial in stage IIB-IV melanoma first published by Andtbacka et al. [59] in 2013.

The notable bystander effect underscores the critical role of a systemic immune response and has driven efforts to further amplify both local and distant therapeutic responses by combining T-VEC with systemic immune checkpoint inhibitors. Chesney et al. [60] published the results of a phase II trial comparing the administration of systemic ipilimumab alone versus the administration of intralesional T-VEC followed by systemic ipilimumab. The results were superior in terms of complete response, partial response and progression-free median survival in the combination treatment group, without a significant increase in adverse effects. This study suggests that patients with BRAF-wild-type tumors had better response rates than those with BRAF-mutant tumors, although these data would require more targeted testing in specifically designed studies. Later, Malvehy et al. [61] carried out another phase II clinical trial with intralesional T-VEC in which they evaluated the non-injected lesions to establish changes in intratumoral CD8+ T-cell density. They noticed that there was an increase in the proportion of infiltrating CD8+ T cells expressing granzyme B and checkpoint markers, including programmed death-1 (PD-1), programmed death ligand 1 (PD-L1) and cytotoxic T lymphocyte antigen-4 (CTLA-4), in uninjected lesions. Additionally, there was a rise in the presence of T helper cells. This study suggests that T-VEC stimulates systemic immune activity and modifies the tumor microenvironment in a manner that is likely to enhance the effectiveness of other immunotherapy agents when used in combination therapy (Table 6).

A recent case has been reported in which a patient who had experienced progression during combination treatment with intralesional T-VEC and systemic pembrolizumab was switched to combination therapy with ipilimumab and nivolumab and, when he again experienced progression, was re-treated with T-VEC monotherapy and showed a significant response that maintained for 6 months [62]. This case illustrates that the modification of the immune response by immune checkpoint inhibitors can stimulate the response to T-VEC.

On the other hand, a report published by Chesney et al. [63] in 2023 that explored the combination of intralesional T-VEC plus pembrolizumab versus placebo plus pembrolizumab did not find any significant difference in terms of overall survival or progression-free survival between the two options evaluated.

Finally, we would like to mention in this paper a phase II trial published by Dummer et al. [64] that explored the possibility of using intralesional T-VEC as a neoadjuvant therapy versus surgery alone in patients with resectable stage IIIB-IVM1a melanoma, reporting a 25% reduction in the risk of recurrence with the use of T-VEC in combination with surgery. This perspective is very interesting, as neoadjuvant therapy in advanced melanoma is an emerging treatment. Oncolytic viruses such as T-VEC present a promising perspective for the future in the treatment of metastatic melanoma alone or in combination with systemic immunotherapies. In addition to the studies mentioned above, several clinical trials whose results are expected to be available in the next few years are ongoing and could potentially change the treatment paradigm for patients with metastatic melanoma.

**Table 6:** Most relevant T-VEC intralesional therapy investigations. Abbreviations: PPR (progression prior to response), CR (complete response), PR (partial response), PFS (progression-free survival) and TTF (time to failure).

Authors	Year of Publication	Type of Study	Target	Treatment	Outcomes	Adverse Effects
Andtbacka et al. [59]	2016	Phase III clinical trial (436 patients)	Skin metastases of melanoma in patients with unresected stage IIIB–IV melanoma.	Intralesional T-VEC versus subcutaneous GM-CSF. A treatment cycle of T-VEC consisted of two consecutive injections (5 weeks for the first cycle and 4 weeks for subsequent cycles). The treatment was administered for a minimum of six months, with no mandatory discontinuation due to disease progression. Based on the findings from a phase II trial, an increase in lesion size or the emergence of new lesions was expected in some patients.	T-VEC produced a decrease in size by ≥50% in 64% of injected lesions, 34% of uninjected non-visceral lesions and 15% of visceral lesions. The complete resolution of lesions occurred in 47% of the injected lesions, in 22% of the uninjected non-visceral lesions and in 9% of the visceral lesions. Of the 48 patients with durable responses, 23 (48%) experienced PPR, including 14 who developed new lesions only. No difference in overall survival was observed, and median duration of response was not reached in patients with PPR versus those without PPR	Fatigue, chills and pyrexia. The only grade three or four adverse events (AEs) observed in ≥2% of the patients receiving T-VEC were cellulitis, occurring in 2.1%. No treatment-related fatal adverse events were reported [65].
Chesney et al. [60]	2018	Phase II clinical trial (198 patients)	Patients with unresectable stages IIIB–IV melanoma.	Talimogene laherparepvec plus ipilimumab or ipilimumab alone. Talimogene laherparepvec treatment began in the first week. Ipilimumab began in the first week in the ipilimumab alone arm and in week 6 in the combination arm.	The objective response rate was notably higher with the combination of talimogene laherparepvec and ipilimumab compared to ipilimumab monotherapy. A total of 38 patients (39% [CR, 13%; PR, 26%]) in the combination therapy group and 18 patients (18% [CR, 7%; PR, 11%]) in the ipilimumab monotherapy group exhibited an objective response. Notably, the responses were not confined to the injected lesions; a reduction in visceral lesions was observed in 52% of the patients in the combination group compared to 23% in the ipilimumab group. The median PFS was 8.2 months in the combination therapy group, compared to 6.4 months in the ipilimumab monotherapy group.	Fatigue, diarrhea, pruritus, rash and nausea were common in both groups. Incidences of grade ≥ 3 AEs were 45% in the combination arm and 35% in the monotherapy arm. The most frequent ones with ipilimumab alone were GI disorders and influenza-like symptoms and lymphopenia in the T-VEC group.
Malvehy et al. [61]	2021	Phase II clinical trial (59 patients)	Patients with unresectable stage IIIB–IV melanoma.	T-VEC was administered via intralesional injection, either with or without ultrasound imaging guidance, following the dosing regimen outlined in the registrational phase III OPTiM study	After a median follow-up of 108.0 weeks, objective/complete response rates in IIIB-IVM1a stages were 28%/14% in the overall population and 32%/18% in the other group. Treatment failure was recorded in 69 (62%) patients. Median TTF was 8.1 months.	Fever, chills, influenza-like illness, nausea, fatigue, injection-site pain, headache, asthenia, arthralgia, vomiting and pain in extremity

### 4.5. Electrochemotherapy

Electrochemotherapy or electroporation is a technique used in the treatment of skin metastases of melanoma and other cutaneous tumors as well as solid tumors skin metastases as it enhances the local tumor-destructive effects of chemotherapy. Under general anesthesia or heavy sedation, a probe array is inserted into the dermal or subcutaneous metastasis for a brief duration. A relatively high-intensity, pulsed electrical current is then applied to the lesion while simultaneously administering a bolus of systemic and/or intralesional chemotherapy, typically bleomycin or cisplatin. The electric current increases the cell membrane permeability and enhances the entrance of the chemotherapeutic drug into the tumor cells. Glass et al. [66] published the results of their first attempts to treat five patients with metastatic melanoma in 1996. They treated them with a palliative purpose with intralesional bleomycin injection followed by electrical pulses, obtaining complete responses in 78% of the tumors and partial response in 17% of them. Their results led to the further investigation of the technique.

There are only a few studies with a strong quality of evidence on electrochemotherapy. Sersa et al. [67] reported a 77% control rate at 124 weeks follow-up in ten patients with tumors treated with intratumoral cisplatin and electric pulses. Later, Gaudy et al. [68] published a randomized controlled study in 2006 that showed good results regarding the use of electrochemotherapy with intralesional bleomycin. Subsequently, in 2017, Kunte et al. [69] reported the results of a series of 151 patients treated mostly with intravenous bleomycin and electrochemotherapy. A complete response was achieved between 58% and 74% of the lesions, although the long-term durability of the response is not reflected in the manuscripts (Table 7).

Electrochemotherapy has been used in solid transplant recipients with subsequent immunosuppression and concomitant advanced melanoma, with favorable outcomes [70]. It has also been useful in the treatment of patients unresponsive to systemic treatment with anti-PD1, triggering an immune response demonstrated by the development of immunomodulated adverse effects and the remission of both treated and untreated lesions with durable complete response of the disease after a follow-up of 18 months [71]. In view of these results, although there are still a few isolated cases, it is hypothesized that electrochemotherapy may have a synergistic effect with systemic immunotherapy. However, the trend towards the development of immunotherapy will probably relegate it to sideline in the future as a palliative approach. It has disadvantages such as poor tolerance to the procedure without general anesthesia or heavy sedation, in addition to the fact that no bystander effect has been detected in untreated lesions.

### 4.6. Other Intralesional Treatments

There are several other intralesional treatments that have been used with different results, and which, when available, may be valid therapeutic alternatives for patients with surgically unresectable cutaneous metastases of melanoma. We believe it is worthwhile to reflect on some of the studies on this subject in this paper.

#### 4.6.1. Interferon-Alpha (IFN-Alpha)

Interferons are glycoproteins with antiviral, antiproliferative and immunomodulatory activity. IFN-alpha is a type I interferon that has been classically used as an adjuvant therapy in advanced melanoma and is also being studied in combination with immune checkpoint inhibitors such as pembrolizumab or ipilimumab [72]. Regarding its intralesional application, Wussow et al. [73] injected 51 patients with metastatic melanoma with promising results, obtaining a local response in 45% of the patients and a systemic response in 18% of them. Thanks to the knowledge we now have about the mechanism of action of the IFN-alpha, the intratumoral administration of dendritic cells generated in the presence of IFN-alpha (IFN-DCs) is currently being investigated as an attempt to improve the intratumoral immune response [74].

#### 4.6.2. Interleukine-12

IL-12 is a molecule produced by monocytes that acts as an inducer of the antitumor response of T lymphocytes and NK cells. The isolated intratumoral administration of recombinant IL-12 in patients with metastatic melanoma has not obtained very satisfactory results [75]. Forms of administration that may obtain better responses are under evaluation [76]. In addition, more studies are needed in this area, since IL-12’s involvement in the immune response can lead to synergistic responses with other treatments, as observed in combination with anti-PD1 treatments in a phase II clinical trial published by Algazi et al. [77].

#### 4.6.3. Ipilimumab

Ipilimumab, an anti-CTLA4 monoclonal antibody which is used as a systemic therapy for the treatment of advanced melanoma, is also being studied as an intralesional therapy. A phase I study by Ray et al. [78] in stage III/IV patients with a combination of IL-2 and intralesional ipilimumab found local response rates in injected lesions in 67% of patients and an abscopal effect in 89%, with good tolerance. This study suggests that treatment with intralesional checkpoint inhibitors may be an available option in the future.

#### 4.6.4. Daromun (1095TIP)

Daromun is an antibody–cytokine fusion protein that combines L19IL2 and L19TNF. A phase II study published by Danielli et al. [79] showed partial responses in 50% of patients and disease stabilization in 25%, with disease progression in the remaining 25%, when used intralesionally in patients with stage III/IV M1a melanoma. It is currently being investigated as a neoadjuvant intralesional therapy combined with or independent of immune checkpoint inhibitors in a phase III clinical trial [80].

## 5. Future Directions

The natural progression of ITM varies, ranging from a slow and relatively benign course with occasional local recurrences to more aggressive advancement characterized by regional and distant metastases. This variability complicates the rational selection of suitable treatment options, such as topical or intralesional immunotherapy, isolated limb infusion or systemic therapy. High local tumor burden and the presence of metastatic disease are consistently identified as factors associated with poor therapeutic response in studies on topical and intralesional treatments for ITM. The detection of prognostic markers in response to intralesional therapies is a pending task. Although new biomarkers would be needed to select for the most appropriate treatment for these patients, either isolated or in combination with other topical, intralesional or systemic treatments, it is also very important to establish when to start each treatment, as the development of neoadjuvant therapies appear promising. Despite the advances made in the treatment of metastatic melanoma, the long-term prognosis remains poor. After reviewing the published evidence and clinical trials in development, it seems that the trend in the future could be to combine systemic immunotherapies with intralesional treatments such as T-VEC [56,81]. Neoadjuvant immunotherapy, both systemic and intralesional, is currently under investigation and could be a strong alternative in the near future [57,82].

## 6. Strengths and Limitations

Although this review covers a large selection of the existing topical and intralesional treatments for advanced cutaneous melanoma, it is not an exhaustive review, and we are aware that there are several other therapeutic alternatives that have not been included in this article. In addition, the comparability of the different published studies is limited, given that the outcomes, target lesions, dosage regimens, previous treatments, follow-up duration, median survival and response rates are not reported uniformly. In addition, when evaluating studies from several years ago, the staging of the patients involved may differ, and the previous treatments that they received may also be different. On the other hand, the quality of the evidence in many studies is weak since they consist of case series, thus limiting the possibility of making general recommendations based on their results. Despite this, we have provided clear guidelines on a topic of relevance for dermatologists and oncologists by conducting an extensive review of the literature. Furthermore, we provide a new perspective on treatments for advanced cutaneous melanoma that are in development in the coming years. We hope to convey to the reader a clear idea of the therapeutic options available for the treatment of advanced cutaneous melanoma in cases in which surgery is not feasible.

## 7. Conclusions

The first-line treatment for cutaneous metastasis of melanoma is surgical excision. In patients that, due to the characteristics of their lesions or because of their comorbidity, are not candidates for surgery, topical and intralesional treatment alternatives can be used. Topical options such as diphenylcyclopropenone, imiquimod or 5-FU have shown effectiveness in thin lesions, limited to the epidermis or dermis, with a higher difficulty to penetrate and be effective in subcutaneous lesions. A few studies have explored different combinations of two topical therapies or topical therapies with intralesional therapies and have shown positive results. The first intralesional immunotherapy developed was BCG injection, but due to its side effects, it has acquired a secondary role, being replaced by other intralesional immunotherapies such as PV-10, IL-2 and T-VEC. There are limitations for the use of intralesional treatments such as the lack of availability and the administration for which ultrasound guidance is sometimes necessary. Many of these intralesional treatments are being combined in different investigations with systemic immunotherapies, with the aim of obtaining synergic responses in those patients with disease refractory to other treatments.

## Figures and Tables

**Table 1:** Selected published studies evaluating the use of DCP as a treatment in cutaneous melanoma, in-transit metastases and cutaneous metastases of melanoma.

Authors	Year of Publication	Type of Study	Target	Treatment	Outcomes	Adverse Effects
Damian et al. [12]	2013	Case series (50 patients)	In-transit metastases and distant skin metastases.	Topical DPC.	Complete clearance in 46% and partial response in 38%. Mean duration of complete response of 17 months (range between 1 and 78 months).	Blistering, regional lymphadenopathy, generalized dermatitis, generalized urticaria and postinflammatory hyperpigmentation.
Veverka et al. [13]	2018	Case series (13 patients)	In-transit metastases.	Topical DPC combined or independent of pulsed-dye laser, dabrafenib, GM-CSF and/or ipilimumab.	Complete regression in 22%, partial regression in 11%, stable disease in 33% and progression in 33%.	Lack of sensitization in one patient.
Read et al. [8]	2017	Case series (58 patients)	In-transit metastases.	Topical DPC.	Complete response in 22%, partial response in 39%, stable disease in 24% and progressive disease in 15%. Mean duration of disease-free interval of 12.3 months and recurrence rate in complete responders of 41%. Median overall survival of 20.9 months.	Exaggerated localized dermatitis.
Lameiras Gibbons et al. [14]	2018	Case series (16 patients)	In-transit metastases	Topical DPC.	Complete response in 37.5%, partial response in 25% and no response in 31.25%.	Local erythema, dose reduction due to intolerance in three patients.
Moncrieff et al. [16]	2016	Case series (35 patients)	In-transit metastases.	Topical DPC.	Complete response in 28.6%, partial response in 31.4% and no response in 40%. Median follow-up of 9 months. No relapses in the group of complete response. 25.7% died of their disease during the follow-up.	Severe eczema leading to dose reduction.
Yeung et al. [15]	2017	Case series (15 patients)	In-transit metastases or unresectable cutaneous melanoma.	Topical DPC.	Complete response in 13%, partial response in 27%, stable disease in 40% and progression in 20%. Mean overall follow-up of 22.7 weeks. Two patients with complete response had no recurrence at 43 and 36 weeks follow-up.	Blistering, dry skin and intermittent pain.
Ci Lo et al. [17]	2020	Comparative study (78 patients)	In-transit metastases.	Topical DPC, isolated limb infusion (ILI) or both.	Mean progression-free survival in ILI group of 18 months compared to 6 months in the DPC-only group. Mean melanoma specific survival of 32 months on DPC, 69 months on ILI and 39 months on dual therapy. Patients who failed to respond to DPC were then treated with ILI which increased their progression-free survival.	Data not reported in the study.

**Table 2:** Most remarkable studies about topical imiquimod and its combinations for the treatment of locoregionally advanced melanoma with cutaneous metastases.

Authors	Year of Publication	Type of Study	Target	Treatment	Outcomes	Adverse Effects
Green et al. [22]	2007	Phase I/II study (13 patients)	Skin metastases of melanoma.	Topical imiquimod plus intralesional IL-2 (3.6 MIU/mL).	Complete response in 46.5% of the lesions, partial response in 9.9% of the lesions, stable disease in 29.1% and progression in 18.1% of the lesions. The lesions with complete clearance did not relapse.	Erythema, discharge from treated lesions, excessive reaction, rigors, local infection, nausea and dyspepsia
Shirakawa García et al. [23]	2011	Case series (3 patients)	Skin metastases of melanoma.	High dose (22 MIU/1.2 ml) intralesional IL-2 plus topical imiquimod 5% plus retinoid cream.	Complete response with no recurrence during treatment (between 4 and 26 weeks), with two patients disease free during a follow-up of 12 and 27 months. One patient had a relapse 6 months after discontinuation but responded well to a new cycle. One developed visceral metastases 18 months after treatment.	Erythema, tenderness, severe pain, ulceration, hyporexia, chills, arthralgias and fatigue
Shi et al. [24]	2015	Case series (11 patients)	Skin metastases of melanoma.	Intralesional IL-2 plus topical imiquimod 5% plus retinoid cream	Complete response with no recurrence after 24 months follow-up in 100%.	Rigors, nausea, asymptomatic hypotension, urticaria and hyporexia
Rivas-Tolosa [30]	2016	Case series (20 patients)	Satellite or in-transit metastases.	Cryotherapy combined with topical imiquimod 5%.	After a mean of 5 sessions, complete response in 40% and partial response in 25%. Systemic disease progressed in 80%.	Erythema and crusting
Teulings et al. [25]	2018	Phase II clinical trial	Skin metastases of melanoma.	Topical imiquimod plus monobenzone. Mean duration of therapy of 16 weeks.	Regression of the lesions in 38% of patients at 12 weeks, which increased to 52% after a longer course of therapy. Median clinical response duration in responding patients of 6 months. Median local progression-free survival of all patients of 13 weeks.	Erythema, rash, pruritus, ulceration, crusting, edema, burning sensation, fatigue, headache, nausea, flu-like symptoms, erysipelas and contact hypersensitivity reaction among others
Sunshine et al. [34]	2020	Case report	In-transit metastases in a renal transplant patient.	Intralesional talimogene laherparepvec (T-VEC) combined with topical imiquimod 5% cream.	Complete clearance of the in-transit metastases when imiquimod was added, with a follow-up of 13 months with no recurrence.	Crusting and locoregional adenopathy
Kibbi et al. [32]	2015	Case series (3 patients)	In-transit metastases.	Intralesional BCG plus imiquimod at the same time or after initiating BCG due to a lack of response or intolerance to the intralesional treatment.	One patient received treatment for 14 months with complete response 47 months after discontinuation. One patient had good initial response but died from an unrelated cause. One patient had a complete response for 6 months since beginning of the treatment and then relapsed.	Inflammation and crusting
Kidner et al. [33]	2012	Case series (9 patients)	In-transit metastases	Intralesional BCG followed by topical imiquimod 5%.	Complete response in 56% and partial response in one patient. Mean follow-up of 35 months with no recurrence in 78% of the patients.	Mild injection pain, injection site reaction, mild fevers and chills
Yeh et al. [35]	2022	Case series (3 patients)	Skin metastases of melanoma	Cryotherapy plus topical imiquimod 5%. The three patients received systemic treatment with ipilimumab, pembrolizumab or nivolumab at the same time or after.	Complete response in 100% with a disease-free period of 6 years.	Local inflammation
Florin et al. [27]	2012	Case series (5 patients)	Skin metastases of melanoma	Topical 5-FU daily in the morning plus topical imiquimod 5% daily at night until a response was seen.	Clinical response in 98% of the lesions treated, with complete response in 56%. Stable lesion in the remaining 2%. After a 6-month follow-up period, one patient relapsed. It is not disclosed in the article if any longer-term relapses occurred.	Erythema, local inflammation and ulceration.

**Table 3:** Trials conducted using intralesional immunotherapy with BCG in advanced melanoma.

Authors	Year of Publication	Type of Study	Target	Treatment	Outcomes	Adverse Effects
Morton et al. [37]	1974	Case series (151 patients)	Skin metastases of melanoma.	Direct injection of BCG into metastatic melanoma lesions limited to the skin and subcutaneous and visceral lesions.	Complete response in 91% of the intradermal lesions injected, and additional regression of uninjected nodules was seen in 17% of the patients. 31% of these remained disease-free from 6 to 24 months. On the other hand, only 31% of the subcutaneous lesions injected regressed.	Fever, chills, localized abscesses, regional lymphadenitis, systemic infection with hepatitis and anaphylactoid reactions.
Cohen et al. [42]	1978	Randomized clinical trial (18 patients)	Skin metastases of melanoma.	Intralesional BCG or intralesional dinitrochlorobenzene (DNCB).	90% of the injected intradermal nodules regressed both with BCG and DNCB. Subcutaneous disease did not respond well. There was no significant difference between BCG- and DNCB-treated patients in terms of disease-free survival and overall survival.	Fever, chills, nausea, major ulceration, cellulitis, distant infection and disseminated intravascular coagulation with BCG. Less adverse effects with DNCB.
Da Gamma Duarte et al. [41]	2018	Phase I clinical trial (NCT01838200). Discontinued due to high-grade immune-related adverse effects.	Stage III/IV metastatic melanoma with skin lesions.	Dose escalation of intralesional BCG followed by up to four cycles of intravenous ipilimumab (anti-CTLA-4).	No evidence of clinical benefit in the five patients treated.	The trial was discontinued following treatment of the first five patients as the two patients that received the escalation dose of BCG developed high-grade immune-related adverse events (irAEs) typical of ipilimumab monotherapy.

**Table 4:** Selected studies concerning intralesional IL-2 treatment. Abbreviations: CR (complete response), PR (partial response) and MIU (million international units).

Authors	Year of Publication	Type of Study	Target	Treatment	Outcomes	Adverse Effects
Radny et al. [45]	2003	Phase II clinical trial (23 patients)	Patients with stage III or IV melanoma, single or multiple skin and soft-tissue metastases.	Interleukin-2 injections were administered intralesionally into the total number of cutaneous and soft-tissue metastases accessible from the skin, 2–3 times weekly, over 1–57 weeks. Single doses varied from 0.6 to 6 MIU depending on the lesion size. The maximum daily dose was 12 MIU of IL-2.	Complete response (CR) of the treated metastases was achieved in 15 patients (62.5%), with the longest remission lasting 38 months to date. In five patients, partial response (PR) was achieved (21%), and in another three patients, progressive disease was observed (one patient not assessable). There were no recurrences in the treated cutaneous lesions previously responding with CR. In stage III disease, the 2-year survival rate was 100%, and the 5-year survival rate was 63%, and in stage IV disease, the 1-year survival rate was 63%, and the 2-year survival rate was 33%.	Dose-dependent inflammatory reaction at the site of injection with local swelling and erythema. Ulceration, fever, flu-like symptoms, fatigue, nausea, mild abdominal pain and gastritis-like symptoms, tachycardia and headache.
Dehesa et al. [46]	2009	Case series (7 patients)	Satellite metastases and/or cutaneous metastases of malignant melanoma in patients with the absence of metastases in other organs confirmed by positron emission tomography.	Interleukin-2 injections twice a week, with a maximum dose per session between 3 and 18 MIU.	Complete response in 95.9%, partial response in 3.7% and no response in 0.4% of the lesions. The duration of the treatment was between 13 and 38 weeks. Smaller lesions located in the epidermis or superficial dermis had better response. In one patient, a subcutaneous lesion had a partial response that was sufficient to allow for surgical excision with clear margins previously not achievable.	Fever, flu-like symptoms, pain, dysgeusia, local infection, bronchospasm, local acrhromia and distant vitiligo. No histopathological differences were observed between the achromic lesions and the normal ones in terms of tumoral–cell viability.
Weide et al. [47]	2010	Phase II clinical trial (51 patients)	Injectable dermal or subcutaneous metastases either in a clinical stage III or clinical stage IV trial.	Intralesional IL-2 3 times weekly. Treatment was terminated when clinical regression and/or necrosis of metastases was evident or if progression occurred that was no longer manageable with the ongoing IL-2 injections. Initial dose of 3 MIU that escalated based on the individual patients’ tolerance. Highest daily dose was 16 MIU.	Complete response rate of 78.7%, partial response rate of 0.7%, 16.3% with stable metastases and 4.3% with progressive lesions. Differences regarding the rate of complete local responses of injected metastases were detected between stage III versus stage IV (96.9% vs. 54.8%, respectively) and the absence or presence of visceral metastases (92.5% vs. 16.5%). Furthermore, efficacy differed significantly between dermal- versus subcutaneous-injected lesions both for stage III disease (CR rate: 97.9% vs. 90.3%, respectively) and stage IV disease (CR rate: 56.7% vs. 34.4%, respectively). No objective responses of non-injected distant lesions were observed. The median follow-up was 25 months. Patients who had stage III disease had higher overall survival rates compared with patients who had stage IV disease (77% vs. 53% after 2 years).	Local swelling and erythema, tumor necrosis, fever, fatigue, nausea, stomach pain, myalgia, headache, itch, dry oral mucosa, hair loss and diarrhea. One patient presented with vitiligo-like depigmentation around the treated metastases.
Khoury et al. [48]	2021	Case series (65 patients)	In-transit and distant skin metastases (50% of the patients with both).	All patients received at least 4 cycles (every 2 weeks) of intralesional IL-2 therapy. The median total IL-2 injected was 33 MIU per patient, with a median of 4 MIU per lesion.	Complete response in 44.6% of patients after 4 cycles. Two patients demonstrated a robust partial response after 4 cycles and received additional intralesional therapy, achieving a complete response. An abscopal effect was noted in 20.7% of patients with complete response. At a median follow-up of 27 months; the disease-free survival was 65.5%, and the overall survival was 69%. The median time to recurrence was 10.5 months.	Mild flu-like symptoms, localized erythema and swelling.

**Table 5:** Results of the main studies with intralesional injection of PV-10 for the treatment of skin metastases of melanoma.

Authors	Year of Publication	Type of Study	Target	Treatment	Outcomes	Adverse Effects
Thompson et al. [52]	2015	Phase II clinical trial (62 patients)	Stage III–IV melanoma with skin metastases.	PV-10 injections into superficial cutaneous and subcutaneous lesions with a volume of 0.5 mL PV-10 per cm^3^ of lesion volume, up to four times over a 16-week period with a follow-up at 52 weeks.	Complete response rate of 26% and overall response rate of 51%. Median time of response of 1.9 months and median duration of response of 4 months. A total of 8% of the patients did not have recurrence after 52 weeks of follow-up, and 26% of the patients experienced complete remission in uninjected lesions.	Transient pain, edema, vesicles, mild or moderate injection site photosensitivity, generalized photosensitivity reaction (1%) mild headache and mild diarrhea. No life-threatening or fatal adverse effects were reported.
Lippey et al. [54]	2016	Case series (19 patients)	Skin metastases of melanoma.	Intralesional injection of PV-10. The number of treatments administered and the timing were individualized. Most patients received only one treatment (63%). Median follow-up of 11.7 months.	Complete response in 26% of the patients, partial response in 26% and stable disease in 11%. Younger patients and those with smaller lesions were more likely to respond to treatment.	Edema, pain, erythema and cellulitis
Read et al. [53]	2018	Case series (45 patients)	Stage III–IV melanoma with skin metastases.	PV-10 injections into superficial cutaneous and subcutaneous lesions with a mean of two PV-10 treatments over a 12-week period. The total dosage of PV-10 was limited to 1500 mg (15 mL of PV-10 solution). Mean follow-up of 21.9 months.	Complete response in 42.2% of the patients, partial response in 44.4%, stable disease in 9.8% and progression in 7.7%. Median time from treatment to best response was 1.1 month. Median time to in-field recurrence was 2.3 months from the best response. Median overall survival from first PV-10 treatment was 25.1 months.	Edema, transient pain, blistering, cellulitis and photosensitivity reactions.

**Table 7:** Comparation of the electrochemotherapy studies published by Gaudy et al. and Kunte et al. Abbreviations: CR (complete response) and PR (partial response).

Authors	Year of Publication	Type of Study	Target	Treatment	Outcomes	Adverse Effects
Gaudy et al. [68]	2006	Randomized controlled study (12 patients)	Skin metastases of melanoma in patients with stage IV melanoma receiving chemotherapy and stage III melanoma without systemic treatment.	Intralesional bleomycin plus electric pulses versus intralesional bleomycin alone.	In a per protocol population, after at least one month follow-up, the overall tumor response (CR + PR) was obtained in 87% of the lesions in the combination arm and in 53% of the monotherapy arm. CR was obtained in 74% and 13%, respectively. The metastases that showed CR did not have any evidence of recurrence at week 12 and 24 post-treatment.	Pain despite local anesthesia, muscle spasms, local erythema, swelling and necrosis.
Kunte et al. [69]	2017	Case series (151 patients)	Skin or mucosal metastases of melanoma.	Intratumoral or intravenous bleomycin plus electric pulses under local or general anesthesia.	A total of 82% of the patients received a single session, 14% received two, 3% received three and 1% received four. Bleomycin was administered via local injection in 13% of the patients and via systemic injection in 87% of the patients. At 60 days after treatment, CR was obtained in 58%, PR in 20%, 20% remained stable and 2% had progression. There was a significant correlation between smaller tumor size and CR.	Local skin reaction, nausea, flu-like symptoms, lymphoedema and post-treatment pain.
